# Novel Bioactive Resin Coating with Calcium Phosphate Nanoparticles for Antibacterial and Remineralization Abilities to Combat Tooth Root Caries

**DOI:** 10.3390/ijms26062490

**Published:** 2025-03-11

**Authors:** Nader Almutairi, Abdullah Alhussein, Mohammad Alenizy, Ibrahim Ba-Armah, Heba Alqarni, Thomas W. Oates, Radi Masri, Gary D. Hack, Jirun Sun, Michael D. Weir, Hockin H. K. Xu

**Affiliations:** 1 Dental Biomedical Sciences PhD Program, Graduate School, University of Maryland, Baltimore, MD 21201, USA; nader.almutairi@umaryland.edu (N.A.);; 2 Department of Biomaterials and Regenerative Dental Medicine, University of Maryland School of Dentistry, Baltimore, MD 21201, USA; 3 Department of Conservative Dental Sciences, College of Dentistry, Prince Sattam bin Abdulaziz University, Alkharj 11942, Saudi Arabia; 4 Department of Restorative Dental Science, College of Dentistry, King Saud University, P.O. Box 60169, Riyadh 11545, Saudi Arabia; 5 Department of Restorative Dental Sciences, University of Hail, Hail 55475, Saudi Arabia; 6 Department of Restorative Dental Sciences, College of Dentistry, Imam Abdulrahman Bin Faisal University, Dammam 31441, Saudi Arabia; 7 Department of Pediatric Dentistry and Orthodontic Sciences, College of Dentistry, King Khalid University, Abha 61421, Saudi Arabia; 8 The ADA Forsyth Institute, Cambridge, MA 02142, USA; jsun@forsyth.org; 9 Center for Stem Cell Biology & Regenerative Medicine, University of Maryland School of Medicine, Baltimore, MD 21201, USA; 10 Marlene and Stewart Greenebaum Cancer Center, University of Maryland School of Medicine, Baltimore, MD 21201, USA

**Keywords:** antibacterial polymers, polymer synthesis, antibiofilm, root caries, coating, remineralization, resin

## Abstract

Tooth root caries account for 10.1% of all dental caries in the USA. This study developed a multifunctional resin coating with calcium (Ca) and phosphate (P) ion release and antibacterial properties to combat root caries. The effects of nano-sized amorphous calcium phosphate (NACP) and dimethylaminohexadecyl methacrylate (DMAHDM) on mechanical, physical, and antibacterial properties against *Streptococcus mutans*, and cytotoxicity on dental pulp stem cells and gingival fibroblasts were evaluated. A coating resin combining urethane dimethacrylate (UDMA), triethylene glycol divinylbenzyl ether (TEGDVBE), DMAHDM, and NACP was synthesized and compared with Seal&Protect and Vanish XT. Experimental groups (UV + 5% DMAHDM + 10%, 15%, and 20% NACP) showed flexural strength (70.9 ± 8.0 to 81.1 ± 6.0) MPa, significantly higher than Seal&Protect (48.2 ± 7.2) MPa (*p* < 0.05) and comparable to Vanish XT (70.2 ± 13.6) MPa, (*p* > 0.05). Elastic modulus (2.2 to 3.3) GPa was lower than Vanish XT (9.4 ± 1.1) GPa (*p* < 0.05). Experimental groups showed an 8 log CFU reduction, 96% reduction in metabolic activity and 87% in lactic acid production, and increased Ca (1.25 ± 0.03) mmol/L and P (0.8 ± 0.001) mmol/L release over 35 days. Cytotoxicity for experimental groups against dental pulp stem cells and human gingival fibroblast was low and matched those of commercial controls already used in clinic. The resin demonstrated potent antibacterial properties, high ion release, low cytotoxicity, and maintained physical and mechanical integrity, offering potential to prevent root caries formation and progression.

## 1. Introduction

The increasing incidence of dental root caries, particularly among the elderly, is a significant public health concern. By 2050, the proportion of elderly individuals in the U.S. population is expected to increase from 12% to 22%. According to a recent study, the untreated root caries in the U.S. population is 10.1% of the total untreated dental caries [1]. The primary reasons are attributed to factors like gingival recession, lower acid resistance of root dentin, reduced salivary flow, and the challenges of managing these lesions. Root dentin exhibits greater susceptibility to demineralization compared to enamel due to its lower mineral content (50%) and a higher critical pH threshold (<6.8), in contrast to enamel’s pH of 5.5 and its high inorganic mineral content (98%). Consequently, root dentin begins to demineralize at a less acidic pH, and its mineral phase, primarily composed of smaller and less densely packed hydroxyapatite crystals, is more prone to dissolution. This increased vulnerability not only exacerbates the risk of caries progression but also complicates the management of root caries [1,2]. It is important to develop dental materials that can effectively mitigate demineralization and inhibit bacterial growth in order to effectively control root caries and enhance long-term results.

Exposed root surfaces are generally managed with periodontal interventions aimed at addressing and treating the causes of recession through various procedures, which are beyond the scope of this paper. However, in many cases, periodontal treatments alone may not fully resolve the issue. Contributing factors such as a patient’s overall systemic health or financial limitations can hinder the success of these periodontal approaches, necessitating alternative or adjunctive treatments to protect the exposed surface. Risk assessment and preventive approaches are always the first line of defense against root caries, which often rely on improving oral hygiene, diet counselling, and fluoride-containing materials due to fluoride’s well-known role in preventing demineralization [3]. In order for the preventive measure to work, it requires patients’ compliance and the presence of high concentrations of fluoride for a long time to give an effect. Other strategies include coating the root surface with materials like glass ionomers or resin-based adhesives. The first option lacks long-term stability, especially in patients experiencing hyposalivation [4], and the latter lacks the potency of antibacterial and remineralization over longer periods of time [5].

Restorative materials incorporating various concentrations of antibacterial monomers such as dimethylaminohexadecyl methacrylate (DMAHDM) have been explored [6]. DMAHDM, a quaternary ammonium compound, damages bacterial cell membranes by facilitating an interaction between the negatively charged bacterial wall and the positively charged quaternary amine in the monomer. This interaction disturbs the electrical balance, ultimately leading to bacterial cell death. It has demonstrated effectiveness against multiple bacteria associated with dental caries and periodontal diseases [7]. Furthermore, research indicates that DMAHDM can preserve its antibacterial activity over extended periods [6].

In addition to the antibacterial monomer, nano-sized amorphous calcium phosphate (NACP) is being increasingly utilized in various dental applications, demonstrating effectiveness in remineralizing demineralized tooth structures [8]. The primary mechanism through which NACP works involves the controlled release of calcium and phosphate ions, essential components for natural remineralization processes. This ion release facilitates the repair of enamel and dentin by reintroducing minerals that have been lost. An important feature of NACP is its “smart” release capability. The release of calcium and phosphate ions increases in acidic environments, which are typically associated with cariogenic conditions. This pH-responsive behavior enhances its effectiveness in promoting remineralization when it is most needed. Studies have shown that NACP can provide long-term release of Ca and P ions. Additionally, NACP-containing materials can be recharged, allowing for sustained ion release over extended periods [9].

Despite the promising results of previous studies on the use of NACP and DMAHDM in resin-based materials, a literature search revealed no report on using these compounds in a UDMA/TEG-DVBE resin to develop a bioactive tooth root coating material. The present study seeks to (1) synthesize a multifunctional resin-based coating that releases calcium (Ca) and phosphate (P) ions while exhibiting antibacterial properties to address root caries, and (2) assess the impact of nano-sized amorphous calcium phosphate (NACP) and dimethylaminohexadecyl methacrylate (DMAHDM) on the mechanical, physical, and antibacterial properties against *Streptococcus mutans*, as well as the cytotoxic effects on dental pulp stem cells and gingival fibroblasts.

## 2. Results

### 2.1. Flexural Strength and Elastic Modulus

**Figure 1:**
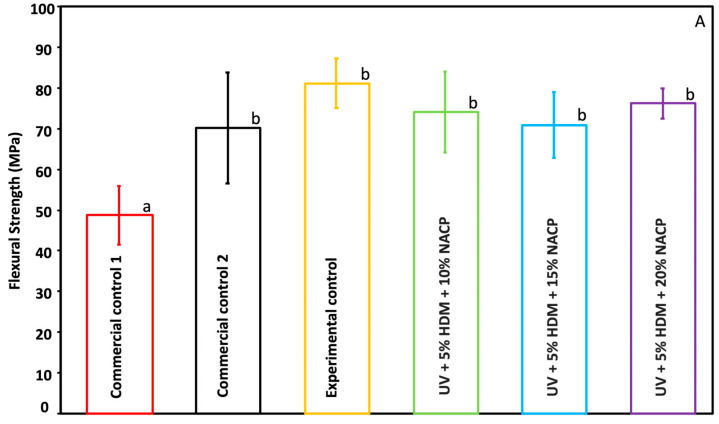

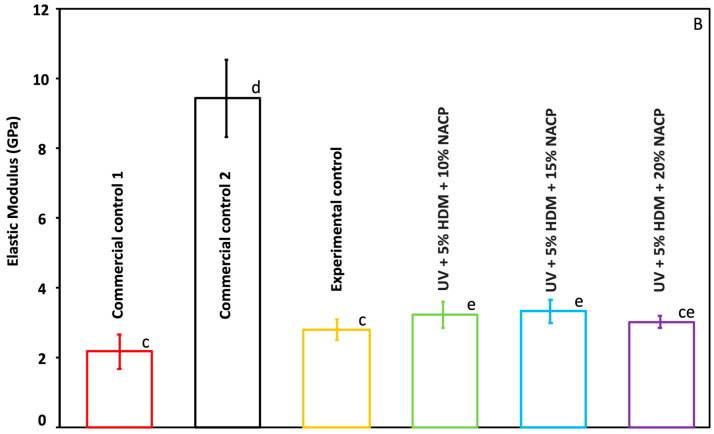
Mechanical properties of commercial controls 1 and 2, experimental control, and experimental groups: (**A**) Flexural strength and (**B**) Elastic modulus (mean ± SD; *n* = 7). Different letters indicate statistically significant differences between the groups (*p* < 0.05).

The flexural strength properties of the coating resin are shown in Figure 1A (mean ± SD; *n* = 7). The experimental control (UV + 0% DMAHDM + 0% NACP), UV + 10% NACP, UV + 15% NACP, and UV + 20% NACP, each with 5% DMAHDM, had a flexural strength of (81.1 ± 6.1) MPa, (74 ± 9.9) MPa, (70.9 ± 8) MPa, and (76.2 ± 3.7) MPa, respectively, showing a significant difference (*p* ≤ 0.05) compared to Seal&Protect (commercial control 1) (48.7 ± 7.2) MPa, while no significant difference was observed between all experimental groups and Vanish XT (commercial control 2) (*p* ≥ 0.05). The elastic modulus values of the different groups are shown in Figure 1B (mean ± SD; *n* = 7). Commercial control 2 showed a significant difference from all other groups at (9.4 ± 1.1) GPa (*p* ≤ 0.05), whereas all experimental groups ranged between (2.7 ± 0.29) GPa and (3.3 ± 0.33) GPa and commercial control 1 at (2.2 ± 0.5) GPa. There was no statistically significant variation observed across all experimental groups (*p* ≥ 0.05).

### 2.2. Degree of Conversion

**Figure 2:**
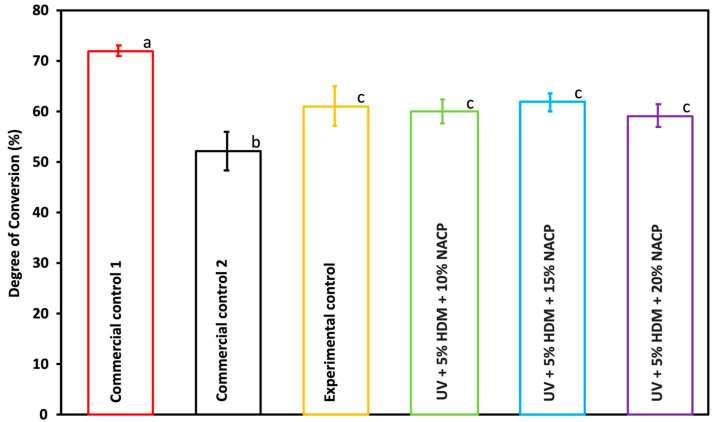
Degree of conversion for commercial controls 1 and 2, experimental control, and experimental groups (mean ± SD; *n* = 3). Groups with different letters indicate statistically significant differences (*p* < 0.05).

Figure 2 demonstrates the degree of conversion (DC) for the coatings. Commercial control 1 was significantly higher compared to all the experimental groups, experimental control, and commercial control 2 (*p* < 0.05). However, all experimental groups, including the control, exhibited a statistically significant difference when compared to commercial control 2 (*p* < 0.05). There was no statistically significant variation observed in the degree of conversion across all experimental groups (*p* ≥ 0.05).

### 2.3. Paste Flowability

**Figure 3:**
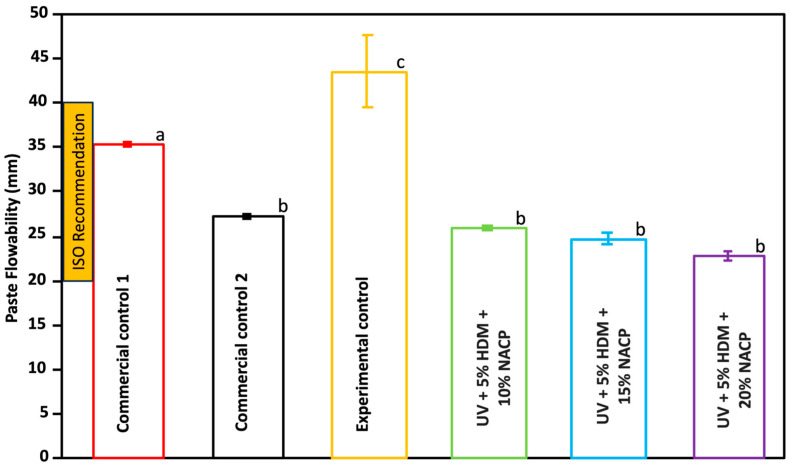
Flowability of the pastes for commercial controls 1 and 2, experimental control, and experimental groups (mean ± SD; *n* = 3). Different letters indicate statistically significant differences between groups (*p* < 0.05).

Figure 3 illustrates the paste flow characteristics of the coating resin (mean ± SD; *n* = 3). The experimental groups did not exhibit any significant differences compared to commercial control 2 (*p* > 0.05). However, both the experimental control and commercial control 1 showed a statistically significant difference compared to all experimental groups and commercial control 2 (*p* < 0.05). All groups mean values were within the ISO standards [10].

### 2.4. Calcium (Ca) and Phosphate (P) Ion Release

**Figure 4:**
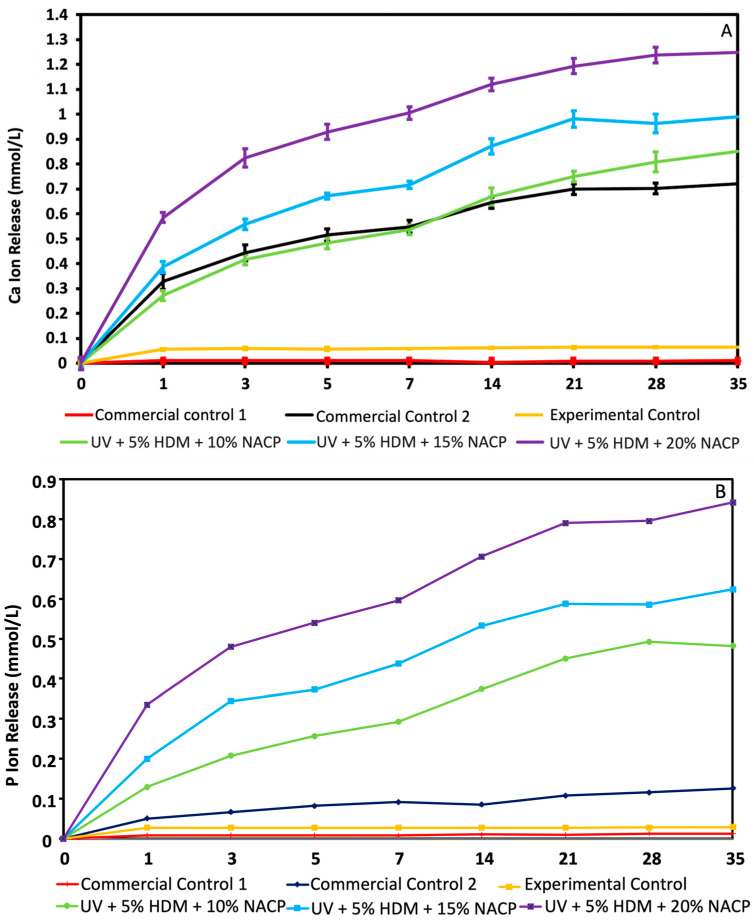
Release of calcium (Ca) and phosphate (P) ions over 35 days (mean ± SD; *n* = 3). (**A**) Calcium ion release and (**B**) phosphate ion release. Groups with different letters indicate statistically significant differences (*p* < 0.05).

Figure 4 presents the release of Ca and P ions from the resin coatings over 35 days (mean ± SD; *n* = 3). The samples containing 10%, 15%, and 20% NACP incorporated into UV resin with 5% DMAHDM showed a consistent increase of Ca (0.852 ± 0.04) mmol/L, (0.99 ± 0.037) mmol/L, and (1.249 ± 0.032) mmol/L and P ion release (0.482 ± 0.0009) mmol/L, (0.62 ± 0.0009) mmol/L, (0.85 ± 0.001) mmol/L, respectively, over the test period. In contrast, neither commercial control 1 nor the experimental control exhibited any detectable ion release. However, during the first two weeks, the Ca ion release from commercial control 2 was comparable to that of the UV resin containing 10% NACP at (0.7 ± 0.02) mmol/L and P concentration of (0.1 ± 0.0002) mmol/L.

### 2.5. Colony-Forming Unit Counts (CFU)

**Figure 5:**
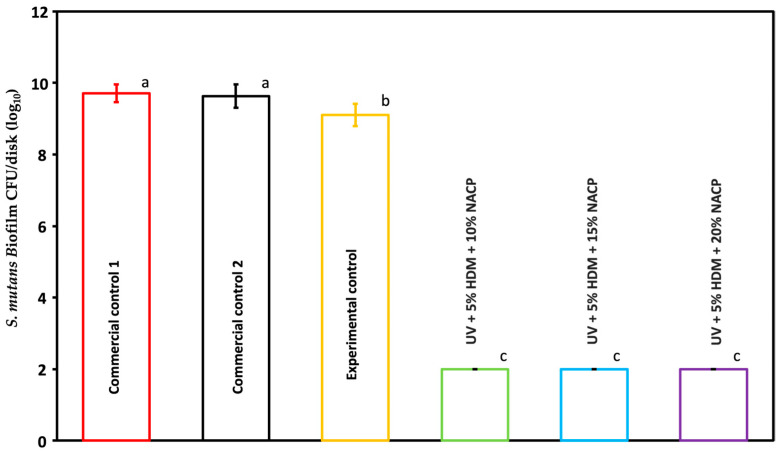
*S. mutans* biofilm colony-forming units (CFUs) per disk (log_10_) (mean ± SD; *n* = 6) for commercial controls 1 and 2, experimental control, and UV + 10, 15, and 20 (%) NACP coating resin. Groups with different letters indicate statistically significant differences (*p* < 0.05).

The CFU counts of 48 h *Streptococcus mutans* biofilms on the coating resin are presented in Figure 5 (mean ± SD; *n* = 6). Incorporating 5% DMAHDM into the resin led to a notable 8 log reduction in CFU counts when compared to both the commercial and experimental control groups (*p* < 0.05). No significant differences in CFU counts were observed among the experimental groups (*p* > 0.05).

### 2.6. Biofilm Metabolic Activity (MTT)

**Figure 6:**
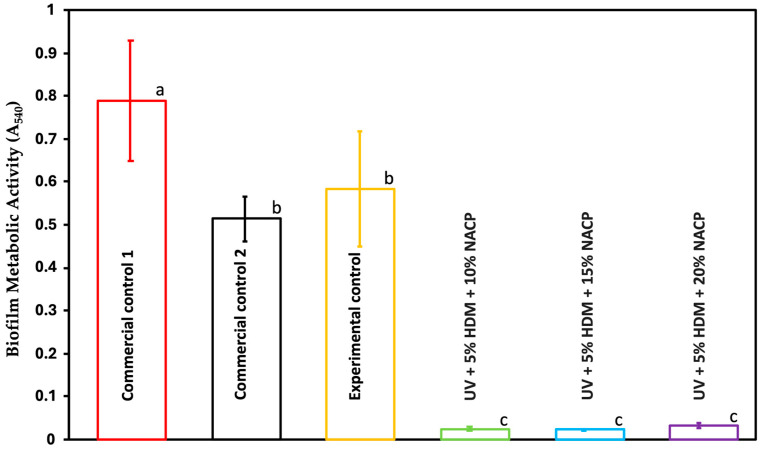
Metabolic activity of the biofilm assessed using the MTT assay for commercial controls 1 and 2, experimental control, and experimental groups (mean ± SD; *n* = 6). Presence of 5% DMAHDM diminished the metabolic activity of the *S. mutans* by ≈96.57%. Groups with different letters indicate statistically significant differences (*p* < 0.05).

Figure 6 displays the metabolic activity of *Streptococcus mutans* biofilm on resin disks, as determined by the MTT assay after 48 h (mean ± SD; *n* = 6). The addition of DMAHDM to the resin resulted in a significant decrease in metabolic activity, around 96%, when compared to both the commercial and experimental control groups (*p* < 0.05). No statistically significant differences were observed among the experimental groups (*p* > 0.05).

### 2.7. Lactic Acid Production (LA)

**Figure 7:**
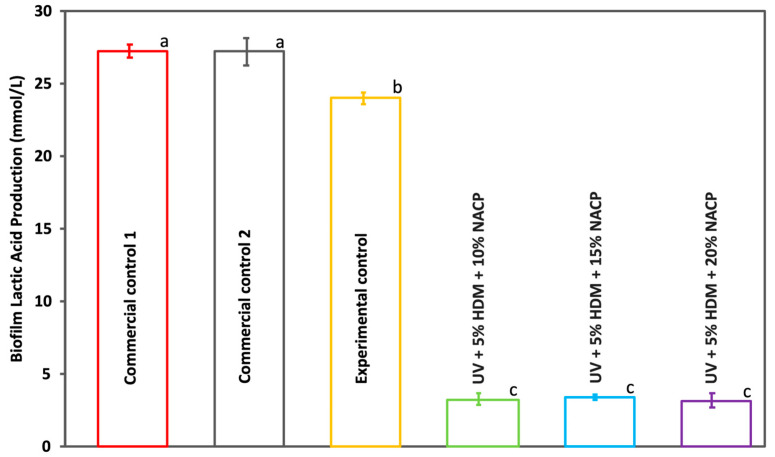
Lactic acid production by *S. mutans* biofilm for commercial controls 1 and 2, experimental control, and experimental groups (mean ± SD; *n* = 6). Groups marked with different letters show statistically significant differences (*p* < 0.05).

Figure 7 shows the (mean ± SD; *n* = 6) lactic acid concentrations produced by *Streptococcus mutans* biofilms. The experimental groups with the UV coating resin containing 5% DMAHDM exhibited a significant 87% reduction in lactic acid production by the bacterial biofilm (*p* < 0.05). In the experimental groups, the mean lactic acid concentration decreased significantly to 3 mmol/L, compared to 27.2 mmol/L in the commercial controls and 24 mmol/L in the experimental control. No statistically significant difference was observed in the LA between the experimental groups (*p* > 0.05).

### 2.8. Cytotoxicity

**Figure 8:**
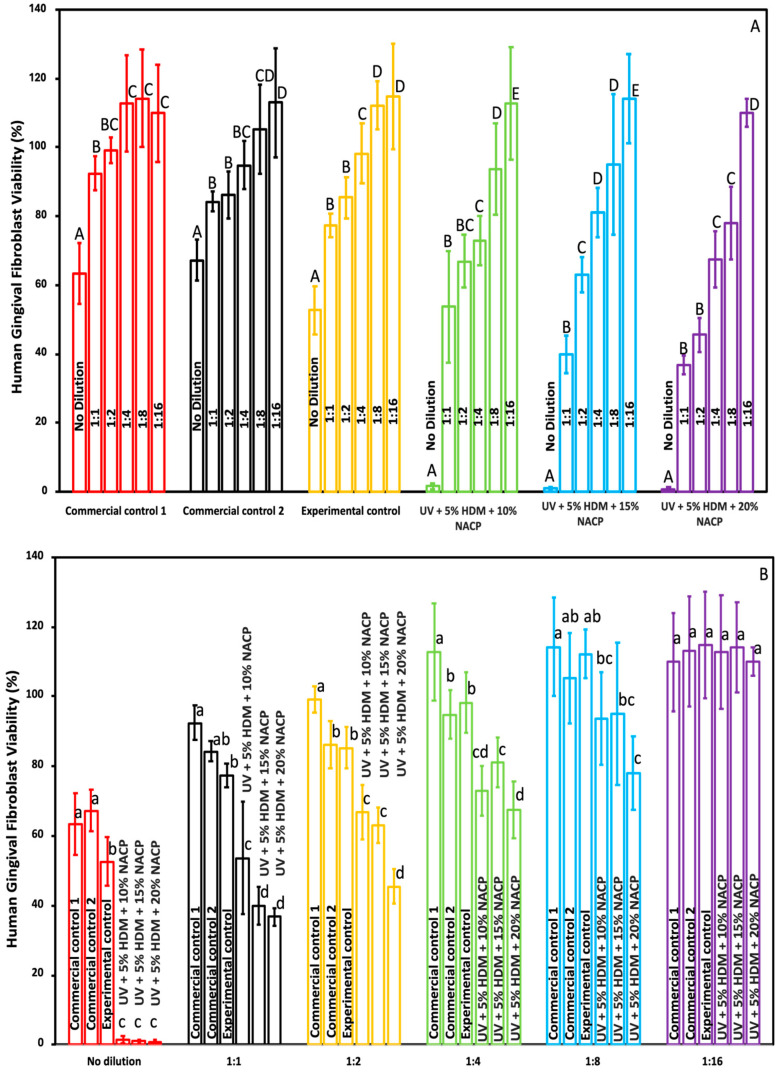
Viability of human gingival fibroblast cells in response to the newly developed coating resin (mean ± SD; *n* = 3 × 3). (**A**) Cell viability for each group and (**B**) cell viability for each dilution. Dilutions in (**A**,**B**) represent the ratio of extract media to fresh fibroblast media: 1:1 = 50 µL:50 µL; 1:2 = 33.3 µL:66.6 µL; 1:4 = 25 µL:75 µL; 1:8 = 12.5 µL:87.5 µL. Groups with different letters indicate statistically significant differences (*p* < 0.05).

**Figure 9:**
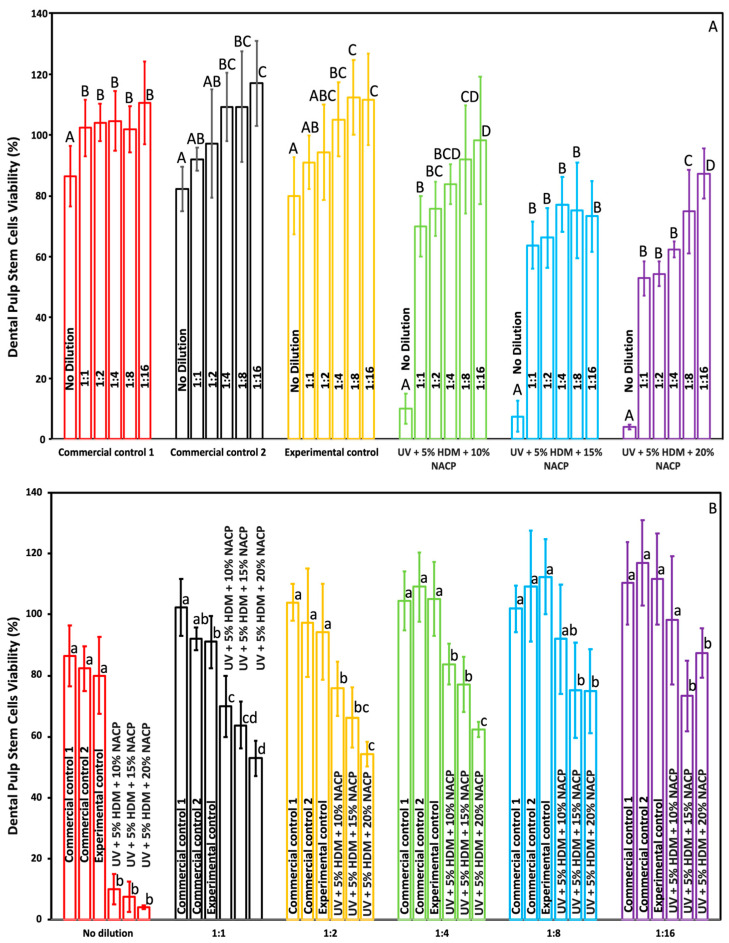
Viability of dental pulp stem cells (DPSCs) in response to the newly developed coating resin (mean ± SD; *n* = 3 × 3). (**A**) Cell viability for each group and (**B**) cell viability for each dilution. Dilutions in (**A**,**B**) represent the ratio of extract media to fresh fibroblast media: 1:1 = 50 µL:50 µL; 1:2 = 33.3 µL:66.6 µL; 1:4 = 25 µL:75 µL; 1:8 = 12.5 µL:87.5 µL. Groups with different letters indicate statistically.

Figure 8 and Figure 9 present the viability of human gingival fibroblasts (HGFs) and dental pulp stem cells (DPSCs) in response to a newly developed resin-based coating material, in comparison to commercial controls (mean ± SD; *n* = 3 × 3). In Figure 8A and Figure 9A, the *x*-axis illustrates the comparison across various dilutions within each group, while Figure 8B and Figure 9B depict the intergroup comparisons at each dilution level. The results indicate that all experimental control groups exhibited significant toxicity when exposed to the undiluted original extract (*p* < 0.05), relative to both commercial and experimental controls. However, a marked reduction in toxicity was observed in the diluted groups. Notably, at a 1:8 dilution, all experimental groups demonstrated acceptable toxicity levels, with cell viability exceeding 75%.

## 3. Discussion

In the present study, a multi-functional root coating resin material was developed by combining a UDMA/TEGDVBE resin base, DMAHDM for antibacterial potency, and NACP for remineralization. Within our experimental groups, incorporating 20% NACP and 5% DMAHDM into the UV resin provided the best calcium and phosphate ion release, antibacterial potency, and low levels of cytotoxicity, while maintaining the mechanical and physical properties. The newly developed coating significantly reduced the biofilm attachment/adhesion, CFU counts by 8 logs, metabolic activity by 96%, and LA concentration by 87%. Meanwhile, it released ions that are deemed necessary for remineralization with levels as high as 1.2 mmol/L for Ca and 0.8 mmol/L for P. This study provides a novel antibacterial and remineralizing resin-based coating to prevent root caries, offering a safe and effective solution to protect exposed root surfaces and enhance preventative dental care.

A previous study found that coatings using adhesives were not fully effective in providing long-term protection against demineralization caused by acid exposure. It was suggested that this might be due to the adhesive coating being too thin to serve as an adequate physical barrier against demineralization, especially after clinical polishing and regular tooth-brushing [11]. The thickness of the coating is influenced by factors such as viscosity and the application process. The inclusion of filler particles in the adhesive can increase its viscosity, thereby enhancing the coating thickness. In this study we included NACP to provide us with the ion release and the same time increase the viscosity of the material.

Coatings used intraorally require good mechanical properties to withstands forces, stresses, and daily brushing routine. The newly developed coating, as seen in Figure 1, demonstrated distinct mechanical properties in comparison to the commercial controls. All experimental groups were significantly higher than commercial control 1 (*p* < 0.05) (48.7 ± 7.2) MPa, but not significantly different from commercial control 2 (*p* > 0.05). This suggests that the addition of NACP, regardless of concentration, produces a flexural strength comparable to certain commercial coatings, yet it surpasses others, potentially enhancing durability. The observed higher flexural strength of commercial control 1 compared to commercial control 2 can be attributed to several key factors in its composition and structure. The incorporation of nanofiller particles 7 nm in size in Vanish XT significantly enhances its mechanical properties. This nanofiller acts as an additional crosslinker and provides intrinsic hardness, strengthening the overall structure. Furthermore, Vanish XT’s resin-modified glass ionomer (RMGI) composition, which includes fluoroaluminosilicate glass, contributes to its improved durability and strength. In contrast, while Seal&Protect also contains nanofillers, its primary focus is on sealing and protecting exposed dentine rather than maximizing mechanical properties. The experimental groups fit between these two commercial controls. The elastic modulus of all experimental groups ranging between (2.7 ± 0.29) GPa and (3.3 ± 0.33) GPa was close but statistically higher than commercial control 1 (2.2 ± 0.5) GPa, indicating a low modulus of elasticity. However, the significantly higher elastic modulus of commercial control 2 (9.4 ± 1.1) GPa compared to both commercial control 1 and the experimental groups points to a stiffer material compared to the other groups. The lack of significant differences among the experimental groups suggests that increasing NACP concentration within the tested range does not markedly influence these mechanical properties, which may allow for formulation flexibility without compromising material performance.

The paste flow characteristics of the coating resin reveal distinct flow profiles between the experimental and control groups. Notably, the experimental coatings with UV resin and NACP concentrations of 10%, 15%, and 20% exhibited no significant difference in paste flow compared to commercial control 2, indicating similar viscosity and handling properties among these formulations. In contrast, both the experimental control and commercial control 1 showed significantly different flow characteristics compared to all NACP-containing experimental groups and commercial control 2. The consistency in flow behavior across the different NACP concentrations within the experimental groups also implies that the addition of NACP, up to 20%, does not negatively impact the material’s ease of application since it is still within the ISO recommendation [10].

The degree of conversion provides insight into the polymerization efficiency of the resin formulations tested. It indicates that commercial control 1 achieved a significantly higher conversion than all experimental groups and commercial control 2, pointing to greater curing efficiency. Among the experimental groups, no significant differences were observed, suggesting that NACP concentrations (10%, 15%, 20%) and 5% DMAHDM do not impact polymerization efficiency. However, all experimental groups differed significantly from commercial control 2, likely due to compositional or curing differences. These findings highlight consistent polymerization across experimental formulations, suggesting potential for optimization in curing. These results align with our previous study [12]. The polymerization degree of vinyl conversion (DC) plays a significant role in determining the mechanical and physical properties of resin-based dental materials. A lower DC can negatively impact these properties and result in the release of unreacted monomers, which may pose a risk of cytotoxicity to surrounding tissues. Generally, the clinically acceptable degree of conversion for dental composites is 55% [13].

The release of calcium Ca and P ions reveals that the experimental coatings with 10%, 15%, and 20% NACP incorporated into UV resin with 5% DMAHDM consistently released Ca and P ions over 35 days in a concentration-dependent manner. This sustained release suggests the potential for prolonged remineralizing effects. Neither commercial control 1 nor the experimental control showed detectable ion release, highlighting the unique capability of NACP-containing formulations to release ions. Notably, during the first two weeks, Ca ion release from commercial control 2 was similar to the UV resin with 10% NACP, though this effect was not sustained, underscoring the potential long-term advantage of NACP-containing coatings.

This study highlights the strong antibacterial properties of the coating resin with 5% DMAHDM against *Streptococcus mutans* biofilms. The CFU counts demonstrate an 8 log reduction in the DMAHDM-containing resin compared to both the commercial and experimental controls, indicating substantial antibacterial efficacy. Similarly, the MTT assay results confirm that DMAHDM incorporation reduces biofilm metabolic activity by 96.57% relative to the controls, highlighting its impact on bacterial viability. In addition to lowering CFU counts and metabolic activity, the DMAHDM-containing resin significantly reduced lactic acid production in the biofilm, decreasing it from 27.2 mmol/L in both commercial controls and 24 mmol/L in the experimental control to 3 mmol/L. Importantly, no significant differences in CFU counts, metabolic activity, or lactic acid production were observed among the experimental groups, suggesting that 5% DMAHDM consistently provides robust antibacterial effect regardless of the filler load. Lactic acid is a primary byproduct of *Streptococcus mutans* metabolism. It is often associated with bacterial virulence; this decrease might indicate reduced pathogenic potential, thereby lowering the likelihood of caries formation [14] The charge density of quaternary ammonium methacrylates (QAMs) in the synthesized resin plays a crucial role in balancing antibacterial efficacy and biocompatibility. A higher positive charge density enhances the antibacterial properties of the resin by increasing electrostatic interactions with negatively charged bacterial cell membranes, leading to effective membrane disruption and bacterial cell death [15].

The viability results for human gingival fibroblasts (HGFs) and dental pulp stem cells (DPSCs), as shown in Figure 8 and Figure 9, assess the cytotoxicity of the newly developed resin-based coating material. In both Figure 8A and Figure 9A, the *x*-axis represents various dilutions, while Figure 8B and Figure 9B allow intergroup comparisons at each dilution level. The undiluted original extract of the experimental resin displayed significant toxicity, with reduced cell viability compared to both commercial and experimental controls (*p* < 0.05). However, this toxicity markedly decreased in diluted samples. At a 1:8 dilution, all experimental groups achieved acceptable cytotoxicity levels, with cell viability exceeding 75%, indicating acceptable biocompatibility and supporting potential clinical application. According to ISO 10993-5, cell viability above 70% indicates that the materials are non-cytotoxic [16]. Previously published work on the biocompatibility of resin-based dental materials to HGFs and DPSCs indicates a similar response. Therefore, we were not anticipating much difference between the two, which is illustrated in Figure 8 and Figure 9. The saliva flow rate for an average individual ranges from 1000 to 1500 mL per 24 h; therefore, utilizing an undiluted extract may not accurately simulate the intraoral environment [17]. In some of the biological experiments, the high standard deviations observed may be attributed to intrinsic differences in metabolic rates, growth patterns, and sensitivities to the resin-based coating. Furthermore, heterogeneity within the cells, such as variations in cell seeding quantities and cycle stages (e.g., cells in proliferation versus quiescence), can result in varying cellular responses [18].

This study has several limitations that should be considered. All experiments were conducted in vitro, which may not fully replicate the complex oral environment, where factors such as saliva, dynamic pH changes, and diverse microbial communities can influence material performance. Additionally, the absence of pH measurements during the biological experiments limits the understanding of the material’s impact on the cellular microenvironment. In vivo studies are necessary to confirm the antibacterial efficacy, ion release, and biocompatibility of the coating under clinically relevant conditions. Additionally, while the resin demonstrated effective ion release and antibacterial properties, the long-term stability of these effects has not been evaluated. Our future paper will assess the durability of the bonding to dentin surface, with more emphasis on long-term ion release and antimicrobial activity as well as abrasion and erosion resistance.

## 4. Materials and Methods

### 4.1. Formulations of the Coating Resins and DMAHDM

The light-cure resin-based coating was synthesized using a 55.8% mass fraction of urethane dimethacrylate (UDMA) (Esstech, Essington, PA, USA) and a 44.2% mass fraction of triethylene glycol divinylbenzyl ether (TEG-DVBE) formulated at a 1:1 molar ratio, as described in a previous study [19]. Camphorquinone (CQ; Aldrich, Saint Louis, MO, USA) of 0.2 wt% was used to initiate photo-polymerization, and 0.8 wt% of ethyl 4-N,N-dimethylaminobenzoate (4EDMAB; Aldrich, Saint Louis, MO, USA) as an accelerator was added to all monomer mixtures. The combination of UDMA/TEG-DVBE is referred to as UV throughout this paper.

The antibacterial monomer DMAHDM was synthesized through a modified Menschutkin reaction, as outlined in prior research [20]. To start, 3 g of ethanol was combined with 10 mmol of 1-bromohexadecane (BHD, TCI America, Portland, OR, USA) and 10 mmol of 2-(dimethylamino) ethyl methacrylate (DMAEMA, Sigma-Aldrich, Saint Louis, MO, USA) in a 20 mL scintillation vial. The mixture was stirred at a constant temperature of 70 °C for 24 h. After the solvent evaporated, DMAHDM was isolated as white, waxy solid material. This was then incorporated into UV resin, creating resin composites containing 5% DMAHDM by weight.

Four concentrations of NACP (0%, 10%, 15%, and 20%) were incorporated into the resin. A spray-drying technique was used to synthesize NACP, as reported previously [21]. Briefly, acetic acid was used to dissolve calcium carbonate and dicalcium phosphate anhydrous to construct a concentrations of 8 mmol/L of calcium (Ca) and 5.333 mmol/L phosphate (P). The resultant molar ratio of Ca/P was 1.5, the same as that for ACP [Ca_3_(PO_4_)_2_], which then was sprayed into a heated chamber of the spray-drying machine. An electrostatic precipitator was used to collect the dry NACP, with an averaged 116 nm particle size. NACP was added to facilitate the release of high levels of Ca and P ions for remineralization and increase the viscosity of the resin while maintaining the mechanical properties. Barium boroaluminosilicate glass particles with a median size of 1.4 µm (Caulk/Dentsply, Milford, DE, USA) silanized with 4% 3-methacryloxypropyltrimethoxysilane were used as a co-filler. All the groups had a filler mass fraction of 20%. Two commercial controls served as commercial controls in this study. Seal&Protect (Dentsply DeTrey GmbH, Konstanz, Germany) with a composition of penta-, di-, and trimethacrylate resins, nanofillers, initiators, a stabilizer, CAHF, acetone, and triclosan. Vanish™ XT Extended Contact Varnish (3M ESPE, St. Paul, MN, USA) consists of a liquid component primarily made up of polyalkenoic acid, HEMA (2-hydroxyethylmethacrylate), water, and initiators (including camphorquinone), along with calcium glycerophosphate. The paste component is a mixture of HEMA, BIS-GMA, water, initiators, and fluoroaluminosilicate glass (FAS glass).

Then, the following groups were formulated:

1. Seal&Protect (designated as “Commercial Control 1”);
2. Vanish XT (designated as “Commercial Control 2”);
3. UV + 0% DMAHDM + 0% NACP + 20% glass (referred to as “Experimental Control”);
4. UV + 5% DMAHDM + 10% NACP + 10% glass (referred to as “UV + 5% HDM + 10% NACP”);
5. UV + 5% DMAHDM + 15% NACP + 5% glass (referred to as “UV + 5% HDM + 15% NACP”);
6. UV + 5% DMAHDM + 20% NACP + 0% glass + (referred to as “UV + 5% HDM + 20% NACP”).

### 4.2. Mechanical and Physical Properties

#### 4.2.1. Flexural Strength and Elastic Modulus

Resin bars for mechanical testing were fabricated using a stainless steel mold with dimensions of 2 × 2 × 25 mm^3^. The specimens were photo-cured for 60 s on each side at a light intensity of 1200 mW/cm^2^ using a Labolight DUO device (GC, Tokyo, Japan). To prevent the formation of an air-inhibited layer, Mylar strips were placed on both sides during curing. Once cured, the samples were stored at 37 °C for 24 h. A three-point bending test (*n* = 6) was conducted to determine the flexural strength and elastic modulus using a Universal Testing Machine (MTS, Insight 1, Cary, NC, USA) with a 10 mm span and a crosshead speed of 1 mm/min [15].

#### 4.2.2. Degree of Conversion

The degree of conversion (DC) was determined using a Fourier-transform infrared (FT-IR) spectrometer (Nicolet 6700, Thermo Fisher Scientific, Waltham, MA, USA), operating over a wavelength range of 400–4000 cm^−1^ with 32 scans and a resolution of 4 cm^−1^. To estimate the remaining methacrylate groups after light exposure, the relative band ratio method was used. This involved analyzing the change in intensity of the C=C vibration peak at 1637 cm^−1^, with the absorbance of the aromatic group at 1583 cm^−1^ serving as an internal reference. For the measurements, a drop of the resin material (mean ± SD; *n* = 3) was placed on the attenuated total reflectance (ATR) crystal, covered with a Mylar strip, and analyzed both before and after light curing for 40 s (Bluephase Style, Ivoclar Vivadent) with a minimum output intensity of 1100 mW/cm^2^, checked with a calibrated radiometer (Cure Rite Visible Curing Light Meter, DENTSPLY Caulk, Milford, DE, USA) [22,23].

#### 4.2.3. Paste Flowability

This test was adapted to meet the ISO 6876/2012 standards [10]. Due to the viscosity of the experimental material, mass measurement was used instead of volume measurement. Specifically, 50 mg of resin from each group (mean ± SD; *n* = 3) was placed on a glass plate measuring 100 × 100 × 3 mm. A second glass plate was carefully positioned on top, followed by a 100 g weight, bringing the total weight to 170 g. After allowing the material to settle at a room temperature of 25 °C for 10 min, the maximum and minimum diameters were measured with a digital caliper (Mitutoyo, Tokyo, Japan). The test was repeated if the difference between the two diameters exceeded 1 mm [24].

#### 4.2.4. Ca and P Ion Release

A sodium chloride (NaCl) solution (133 mmol/L) was prepared using deionized water and adjusted to pH 4 with 50 mmol/L lactic acid. Similar to prior studies, three specimens measuring 2 × 2 × 12 mm were submerged in 50 mL of the solution, resulting in a specimen-to-solution volume ratio of 2.9 mm^3^/mL, which is comparable to approximately 3.0 mm^3^/mL in earlier research [25]. The concentrations of calcium (Ca) and phosphate (P) ions were monitored at 1, 3, 7, 14, 21, 28, and 35 days. At each time point, 0.5 mL of solution was removed and replaced with fresh solution. The Ca and P concentrations were determined using a microplate reader (SpectraMax M5, Molecular Devices, Sunnyvale, CA, USA) with known standards and calibration curves. The calibration curves were generated using five calcium standards (0.08, 0.16, 0.24, 0.32, and 0.4 mmol/L) and five phosphate standards (0.008, 0.016, 0.024, 0.036, and 0.048 mmol/L). Ion release was reported as cumulative concentrations. Additionally, the pH of the solution was routinely monitored and adjusted back to pH 4 using 50 mM lactic acid.

### 4.3. Streptococcus mutans (S. mutans) Biofilm Model

#### 4.3.1. Resin Samples for Biofilm Testing

Resin discs, with a diameter of 8 mm and a thickness of 1 mm, were prepared (*n* = 6). Each sample underwent photopolymerization for 60 s at 1200 mW/cm^2^ using a Labolight DUO device (GC, Tokyo, Japan), with exposure on both sides, followed by storage at 37 °C for 24 h. Afterward, the samples were immersed in distilled water and agitated for one hour to help remove any unreacted monomers [25]. The resin discs were then sterilized using ethylene oxide (Anprolene AN 74i, Andersen, Haw River, NC, USA) and subjected to a seven-day degassing process according to the manufacturer’s instructions to remove any remaining ethylene oxide [26].

#### 4.3.2. Bacterial Inoculation and Biofilm Formation

The use of bacterial species in this study was approved by the Institutional Review Board at the University of Maryland, Baltimore (HP-00052180). *Streptococcus mutans* (UA159), a bacterium known for its role in dental caries, was chosen for the experiment. The bacteria were cultured in brain heart infusion (BHI) broth (Sigma-Aldrich, St. Louis, MO, USA) at 37 °C with 5% CO_2_ for 16 to 18 h. The inoculum concentration was adjusted to 10^7^ colony-forming units (CFUs/mL) using a spectrophotometer (Genesys 10S, Thermo Scientific, Waltham, MA, USA), referencing a standard curve of optical density at 600 nm (OD600) against CFU/mL. Each resin disc was placed in a well of a 24-well plate and covered with 1.5 mL of BHI culture media containing 2% sucrose. The plate was incubated for 24 h. After this period, the composite disks were transferred to fresh 24-well plates containing 1.5 mL of new media with sucrose and incubated for another 24 h.

#### 4.3.3. Biofilm Colony-Forming Unit Counts

The biofilm-coated resin discs (*n* = 6) were placed into a 24-well plate containing 1 mL of phosphate-buffered saline (PBS). The biofilms were then harvested using a combination of scraping and sonication/vortexing. The resulting bacterial suspensions were serially diluted from 10^1^ to 10^6^ and plated onto BHI agar plates. These plates were incubated for 48 h at 37 °C with 5% CO_2_. After the incubation period, colonies were counted using a loupe (Reichert Quebec Darkfield Colony Counter, Depew, NY, USA), and the biofilm colony-forming units (CFUs) were determined by multiplying the colony count by the dilution factor [27].

#### 4.3.4. Biofilm Metabolic Activity

The metabolic activity of the biofilm was assessed using a 3-[4,5-dimethylthiazol-2-yl]-2,5-diphenyltetrazolium bromide (MTT) assay (WST-8, Selleckchem, Houston, TX, USA). The resin-coated samples, along with their adhered biofilms, were placed into a 24-well plate containing 1 mL of MTT dye at 0.5 mg/mL concentration in PBS. The samples were incubated for 1 h at 37 °C with 5% CO_2_. After incubation, each disc was transferred to a new 24-well plate containing 1 mL of DMSO per well and kept at room temperature (25 °C) in the dark for 20 min. To measure absorbance, 200 µL of the DMSO solution was transferred to each well of a 96-well plate, and absorbance was measured at 540 nm using a microplate reader (SpectraMax M5, Molecular Devices, Sunnyvale, CA, USA). Higher absorbance values reflected increased metabolic activity in the biofilm. This procedure was repeated three times [28].

#### 4.3.5. Lactic Acid Production by Biofilms

After 48 h of biofilm attachment, the resin-based coating samples were transferred to 24-well plates containing buffered peptone water (BPW, Aldrich, St. Louis, MO, USA) with 0.2% sucrose. The samples were incubated at 37 °C for 3 h in a 5% CO_2_ atmosphere. Lactate levels in the BPW were measured using a microplate reader (SpectraMax M5, Molecular Devices, Sunnyvale, CA, USA) and quantified by the lactate dehydrogenase enzyme, which determined the optical density at 340 nm, as previously outlined. This procedure was performed in triplicate [29,30].

### 4.4. Human Gingival Fibroblast (HGF) and Dental Pulp Stem Cells Cytotoxicity

A cytotoxicity assay was performed using human gingival fibroblasts (hGFBs, CRL-4061, ATCC) and dental pulp stem cells (DPSC, PT-5025, Lonza, Basel, Switzerland), following approval from the University of Maryland. hGFBs were cultured in fibroblast medium (FM) containing 2% fetal bovine serum, 10,000 units/mL of penicillin, and 10,000 µg/mL of streptomycin. DPSCs at passage 6 were cultured in DPSC basal medium (PT-5025, Lonza, Basel, Switzerland), supplemented with 2 mM L-glutamine, 2% fetal bovine serum, 100 mM ascorbic acid solution, GA-1000, 100 IU/mL penicillin, and 100 IU/mL streptomycin. Once cell viability exceeded 90%, cells were seeded in a 96-well plate at a density of 5000 cells per well. Resin disks were prepared using a 4 mm diameter and 1 mm thickness mold. The disks were sterilized using ethylene oxide gas and degassed for seven days.

Eluents were prepared by immersing each resin disk in 4 mL of medium for 24 h at 37 °C. The surface area to solution volume ratio was 0.63 cm^2^/mL, which aligns with the ISO-recommended range of 0.5–6 cm^2^/mL. Biochemical assays were performed on the initial extract solutions and their serial dilutions (1:1, 1:2, 1:4, 1:8, and 1:16) to assess cytotoxicity for potential in vivo use. hGFB cells were exposed to 100 µL of the initial extracts and diluted solutions for 24 h, with culture media without extracts used as a negative control. Cell viability was assessed using a Cell Counting Kit-8 (CCK-8). After 24 h of incubation in a 96-well plate, 10 µL of CCK-8 solution was added to each well. The plate was further incubated for 2 h at 37 °C with 5% CO_2_. Absorbance at 450 nm, reflecting live cell dehydrogenase activity, was measured using a plate reader (SpectraMax M5, Molecular Devices, Sunnyvale, CA, USA). Cell viability was expressed as a percentage relative to the control group.

### 4.5. Statistical Analysis

The assumptions of normality and equality of variances were verified using the Shapiro–Wilk test and Levene’s test, respectively. The factors considered in the analysis were the incorporation of DMAHDM at two levels (0% and 5%) and NACP at four levels (0%, 10%, 15%, and 20%). Data were analyzed using one-way analysis of variance (ANOVA) followed by Tukey’s post hoc test for pairwise comparisons (SigmaPlot 12.0; SYSTAT Software Inc., San Jose, CA, USA). Statistical significance was pre-set at α = 5%.

## 5. Conclusions

This study demonstrates that the newly developed resin-based coating material, incorporating 20% NACP and 5% DMAHDM, offers promising mechanical, antibacterial, ion release, and biocompatibility properties for exposed root surfaces. The experimental coatings showed flexural strength and elastic modulus comparable to commercial controls. Furthermore, the sustained release of calcium and phosphate ions from the coating resin underscores its potential for long-term remineralization, a characteristic absent in commercial alternatives. The incorporation of 5% DMAHDM significantly inhibited *Streptococcus mutans* biofilm growth, metabolic activity, and lactic acid production, reducing CFU counts by 8 log, which is essential for caries prevention. Biocompatibility testing with human gingival fibroblasts and dental pulp stem cells showed initial cytotoxicity in undiluted extracts, but this was significantly reduced with dilution; at a 1:8 dilution, cell viability exceeded 75%, indicating acceptable biocompatibility and supporting potential clinical application. Altogether, these results suggest that the experimental resin-based coating, with its combined remineralizing and antibacterial effects, may effectively protect against caries and biofilm formation while remaining biocompatible with oral tissues. This study provides a novel antibacterial and remineralizing resin-based coating to prevent root caries, offering a safe and effective solution to protect exposed root surfaces and enhance preventative dental care.

## Data Availability

The raw data supporting the conclusions of this article will be made available by the authors on request.
